# The importance of distinguishing pseudogenes from parental genes

**DOI:** 10.1186/s13148-014-0033-5

**Published:** 2014-12-31

**Authors:** Luke B Hesson, Robyn L Ward

**Affiliations:** Adult Cancer Program, Lowy Cancer Research Centre and Prince of Wales Clinical School, UNSW, Sydney, Australia

## Dear Editor,

The July-August 2014 issue of *Clinical Epigenetics* featured a research article describing *PTEN* promoter hypermethylation in multiple myeloma by Piras *et al*. [1]. The importance of the *PTEN* gene has resulted in significant efforts to identify sequence, expression and methylation changes in cancer. Piras *et al*. concluded that *PTEN* hypermethylation occurred in a subset of multiple myeloma cases but that hypermethylation did not correlate with reduced gene expression or clinical parameters. The *PTEN* mRNA shares 97.8% sequence identity with a pseudogene known as *PTENP1*. A 921-bp region of the promoters of these genes is also 91% identical. Consequently, careful consideration of assay design is required to avoid amplification of *PTENP1* rather than *PTEN* sequences. However, the method used by Piras *et al*. for measuring *PTEN* mRNA did not distinguish between these homologues, despite numerous studies showing that *PTENP1* mRNA is ubiquitously expressed in both normal and cancer specimens [2–5]. Furthermore, previous studies have demonstrated that apparent methylation of the *PTEN* promoter is likely attributable to the non-specific amplification of the highly homologous *PTENP1* gene [6,7]. We have shown that the only reliable method for distinguishing between *PTEN* and *PTENP1* promoter methylation is single-molecule bisulfite sequencing that utilizes sequence differences between the two genes to separately analyze individual promoter molecules [6,8]. These methodological challenges make comparisons between methylation and expression impossible when using assays that do not reliably discriminate between *PTEN* and *PTENP1*, and also negate the value of correlating these features with clinicopathological characteristics.

The challenges posed by sequence homology with pseudogenes are by no means particular to the *PTEN* gene. For example, the DNA mismatch repair gene *PMS2* shares >95.2% sequence identity with at least six other genes (*PMS2CL*, *PMS2L2*, *PMS2P4*, *PMS2P5*, *PMS2P1* and *PMS2P11*) making analysis of the *PMS2* CpG island promoter region particularly challenging.

In light of the recent manuscript by Piras *et al*., it is necessary to highlight the importance of rigorous methodology when investigating DNA methylation changes in cancer, especially concerning genes with homologues or pseudogenes such as *PTEN*.
